# NCI 10211: a phase II, single-arm study of berzosertib in combination with irinotecan in patients with advanced *TP53* mutant gastroesophageal cancer

**DOI:** 10.1093/oncolo/oyaf400

**Published:** 2025-12-03

**Authors:** Margaret C Wheless, Shannon Stockton, Heloisa P Soares, Farshid Dayyani, Anwaar Saeed, Edward Kim, Ning Jin, George Yacoub, Vaia Florou, Gregory D Ayers, Bella S Guerrouahen, Alejandro Contreras, Li Li, Katherine V Ferry-Galow, Steven Gore, Satya Das, Jordan Berlin

**Affiliations:** Vanderbilt University Medical Center/Vanderbilt-Ingram Cancer Center, Nashville, TN 37232, United States; Florida Cancer Specialists and Research Institute, Largo, FL 33770, United States; Division of Oncology, University of Utah Huntsman Cancer Institute, Salt Lake City, UT 84112, United States; University of California Irvine Chao Family Comprehensive Cancer Center, Orange, CA 92868, United States; Department of Medicine, University of Pittsburgh Medical Center/Hillman Cancer Center, Pittsburgh, PA 15261, United States; UC Davis Comprehensive Cancer Center, Sacramento, CA 95817, United States; Department of Medicine, Ohio State University Comprehensive Cancer Center, Columbus, OH 43210, United States; Atrium Health Wake Forest Baptist, Winston-Salem, NC 27157, United States; Division of Oncology, University of Utah Huntsman Cancer Institute, Salt Lake City, UT 84112, United States; Vanderbilt University Medical Center/Vanderbilt-Ingram Cancer Center, Nashville, TN 37232, United States; The University of Texas MD Anderson Cancer Center, Houston, TX 77030, United States; The University of Texas MD Anderson Cancer Center, Houston, TX 77030, United States; Frederick National Laboratory for Cancer Research, Frederick, MD 21701, United States; Frederick National Laboratory for Cancer Research, Frederick, MD 21701, United States; National Cancer Institute, Bethesda, MD 20892, United States; Phanes Therapeutics, San Diego, CA 92121, United States; Vanderbilt University Medical Center/Vanderbilt-Ingram Cancer Center, Nashville, TN 37232, United States

**Keywords:** gastroesophageal cancer, DNA damage repair, TP53 mutation, ATR inhibition

## Abstract

**Background:**

Mutations in *TP53* are common in gastroesophageal cancer and portend a poor prognosis. Tumor cells with *TP53* mutations increasingly rely on ataxia-telangiectasia and Rad3-related (ATR) protein to respond to and repair DNA damage induced by cytotoxic chemotherapy. We aimed to assess the efficacy of ATR inhibitor, berzosertib, with irinotecan in patients harboring *TP53*-mutated, metastatic gastroesophageal cancer.

**Methods:**

NCI 10211 is a phase II trial that enrolled patients with *TP53*-mutated, unresectable or metastatic gastroesophageal adenocarcinoma to receive berzosertib with irinotecan on day 1, 15 in 28-day cycles. Initially, patients who had progressed on at least 1 prior line of therapy were enrolled which was later amended to at least 2 prior lines of therapy. The primary outcome was objective response rate (ORR), and secondary outcomes included progression-free survival (PFS) and overall survival (OS). Nine patients underwent biopsy for correlative studies, which included assay evaluation of γH2AX, NBS1, and KAP1 p-Ser 824 expression.

**Results:**

Of the 17 patients enrolled, 16 were evaluable for the primary endpoint of ORR. The ORR was 0%, disease control rate (DCR) of 56.2%, median PFS (mPFS) of 4.01 months, and median OS (mOS) of 6.21 months. The study did not meet its primary endpoint. The most common treatment-related adverse events were nausea (52.9%), anemia (41.2%), diarrhea (41.2%), and lymphopenia (41.2%) without any unexpected adverse events.

**Conclusion:**

This novel combination of ATR inhibitor berzosertib with irinotecan did not lead to objective responses in patients with *TP53*-mutated, advanced gastroesophageal adenocarcinoma. The combination regimen was well tolerated without unexpected adverse events. This trial was registered with ClinicalTrials.gov (NCT03641313).

Lessons Learned

- Combination berzosertib and irinotecan did not lead to objective responses in *TP53*-mutated, metastatic gastroesophageal cancer in the second- or later-line setting.
- The DCR of 56.2% is higher than historical later-line chemotherapy options and no new safety events were observed.
- ATR inhibitors could be more effective in patients whose tumors exhibit ATM loss or in combination with other therapies such as immunotherapy in patients whose tumors harbor DNA damage repair deficiencies.

## Introduction

Gastroesophageal cancer (GEC) has been increasing in prevalence in Western countries and remains a significant cause of worldwide mortality with 5-year survival rates between 5% and 8% in the metastatic setting.^1^^,^^2^ Patients with GEC, comprised of esophageal adenocarcinoma (EAC), gastroesophageal junction adenocarcinoma (GEJAC), and gastric adenocarcinoma (GC) often present with advanced disease and are treated with combination chemotherapy, targeted therapy, and immunotherapy when eligible.^3^ Unfortunately, the median overall survival (mOS) for metastatic or advanced disease in the first-line setting is just 12 months, highlighting the need for innovative and effective treatments for these patients.^4^^,^^5^

Chemotherapy, such as oxaliplatin or irinotecan, works by inducing DNA damage.^6^^,^^7^ Resistance arises when cancer cells develop efficient DNA damage repair mechanisms by activating regulators of DNA damage response, including ataxia-telangiectasia mutated (ATM) and ataxia-telangiectasia and Rad3-related (ATR) proteins.^6^^,^^8–10^ ATR and ATM induce cell cycle arrest via p53 activation and promote DNA repairs.^11^ Solid tumors commonly have aberrant ATM activity, leading to increased reliance on ATR.^12^^,^^13^ In cells with *ATM* mutation, dysregulated ATM signaling, p53 loss, or *TP53* mutation, ATR serves as the major mechanism to induce response to DNA damage.^6^ Targeting ATR to dampen DNA damage repair induced by chemotherapy has been hypothesized to increase response in patients treated with DNA damaging chemotherapy. In GEC, *TP53* mutations have been reported in more than 50% of cases,^14^ which can ultimately cause *TP53*-mutated cancer cells to rely on ATR activity for DNA damage repair induced by chemotherapy, like irinotecan.

Other markers of DNA damage such as γH2AX, a phosphorylated histone, accumulate at sites of DNA double-stranded breaks.^15^^,^^16^ γH2AX co-localizes with KAP1, a target of ATM phosphorylation after DNA damage. Because KAP1 is not dependent on ATR for phosphorylation, it serves as a marker of DNA damage in ATR inhibition.^17^ NBS1, with γH2AX, recruits and activates DNA repair mechanisms.^18^ Together, these markers correlate with DNA damage response in patients treated with ATR inhibitors like berzosertib. Berzosertib demonstrated preclinical and clinical activity as monotherapy and with chemotherapy in several solid tumors in phase I and II trials.^6^^,^^19–21^ Based on the rationale that ATR inhibition with berzosertib in combination with topoisomerase inhibitors may have a synergistic effect, patients with *TP53*-mutated GEC received berzosertib with irinotecan in this phase II trial to evaluate efficacy by objective response.

## Trial information

This was a single-arm, multicenter, phase II clinical trial. Patients received berzosertib 270 mg/m^2^ IV and irinotecan 180 mg/m^2^ IV on days 1, 15 every 28 days based on the results from the phase I NCI 9938 trial.^22^ The first 6 patients were part of the safety lead-in, lasting 28 days. During the 28-day period while dose-limiting toxicities were observed, further enrollment was halted. Planned research biopsies were performed in patients enrolled after the safety lead-in period and collected 24 hours (±3 hours) post–irinotecan on C1D2 and 24 hours (±3 hours) post–berzosertib on C2D2. This protocol (see Supplement 1 for protocol information—see online supplementary material for protocol information) was designed collaboratively with the National ­Cancer Institute (NCI) Cancer Therapy Evaluation Program (CTEP) and registered with ClinicalTrials.gov (NCT03641313). All patients provided informed consent after the trial was approved by the NCI central institutional review board.

Adult patients with metastatic or unresectable GEC and measurable disease were eligible if their tumor harbored a *TP53* mutation on exons 2 or 4-11. These *TP53* exons were chosen since 95% of all mutations leading to p53 inactivation are located on exons 5-8 with the other exons encompassing the majority of the other inactivating hotspot mutations, to broadly include all patients with an inactivating *TP53* mutations.^23^ The original version of the protocol included patients who had progressed on at least 1 line of therapy; the protocol was later amended to include only patients who had received at least 2 prior lines of therapy (Table 1). Archival tissue analysis for *TP53* status could be conducted via any next-generation sequencing assay (done at a Clinical Laboratory Improvement Amendments [CLIA]-certified laboratory) and was reviewed and confirmed by the study principal investigator prior to patient registration. Patients whose tumors are HER2-positive must have progressed on trastuzumab/chemotherapy, while patients whose tumors are microsatellite instability-high (MSI-H) must have received prior pembrolizumab. ECOG performance status was restricted to ≤1 (Karnofsky ≥ 60%). Adequate organ and marrow function for enrollment was defined as WBC ≥3000/mcL; ANC ≥1500/mcL; platelets ≥100 000/mcL; hemoglobin ≥ 9 g/dL; total bilirubin within normal institutional limits; AST/ALT ≤ 2.5 × institutional upper limit of normal, if liver involvement ≤ 5x ULN; creatinine clearance ≥45 mL/min/1.73 m^2^ for patients with creatinine levels above institutional normal. Following the safety run-in, the first 9 patients enrolled must have been willing to undergo endoscopic or image-guided tumor biopsies for correlative studies. Exclusion criteria included early-stage or resectable GEC, prior radiation or chemotherapy within 4 weeks, prior irinotecan, and untreated or symptomatic brain metastases.

**Table 1.** Trial information—NCT03641313.

**Disease**	*TP53*-mutated (exons 2 or 4-11) gastroesophageal cancer
**Stage of disease/treatment**	Metastatic or unresectable
**Prior therapy**	At least 1 prior regimen
**Type of study**	Phase II
**Primary endpoints**	Objective RECIST response based on patient’s best response that is documented during protocol therapy
**Secondary endpoints**	Overall survival, progression-free survival, duration of response, time to progression

## Drug information

Patients received berzosertib 270 mg/m^2^ IV (Table 2—A) and irinotecan 180 mg/m^2^ IV on days 1, 15 every 28 days (Table 2—B). The first 6 patients enrolled were part of the safety lead-in which lasted 28 days. Those enrolled in the safety lead-in were observed for dose-limiting toxicity, defined as grade 4 hematologic toxicity lasting ≥7 days, febrile neutropenia with ≥grade 2 fever and grade 4 neutropenia, grade 4 ­diarrhea despite supportive measures, and any other non-hematologic toxicity ≥grade 3. Dose modification of either agent was allowed based on toxicities possibly, probably, or definitely related to therapy. The maximum allowed treatment interruption was 4 weeks. If 1 agent was interrupted or discontinued, patients could continue monotherapy with the other agent. Restaging computed tomography (CT) scans were obtained every 2 months. Patients were continued on therapy until disease progression, illness preventing further treatment, unacceptable toxicities, patient withdrawal from study, or study termination. After the final dose of irinotecan or berzosertib, patients were monitored for toxicity at least 30 days.

**Table 2.** Drug information.

A	
**Generic/working name**	Berzosertib
**Company name**	Merck
**Drug type**	Targeted therapy
**Drug class**	ATR inhibitor
**Dose**	270 mg/m^2^
**Route**	IV
**Schedule of administration**	Days 1 and 15 of 28-day cycle

B	
**Generic/working name**	irinotecan
**Company name**	Pfizer
**Drug type**	chemotherapy
**Drug class**	Topoisomerase I inhibitor
**Dose**	180 mg/m^2^
**Route**	IV
**Schedule of administration**	Days 1 and 15 of 28-day cycle

Patients were assessed prior to administration of therapy with each cycle and were permitted to proceed with therapy if platelets were ≥100 000/mcL, ANC ≥1000 m/mcL, creatinine was normal or ≥45 mL/min/1.73 m, bilirubin was normal, AST/ALT ≤2.5 × institutional upper limit of normal (or ≤5x ULN if liver involvement), and potassium was within institutional normal limits (or corrected with repletion). Dose modifications of irinotecan or berzosertib were allowed if toxicity was related to therapy. Once dose-reduced, re-escalation was not allowed. Dose modifications for nausea, vomiting, diarrhea, neutropenia, and thrombocytopenia were based on the grade of toxicity.

## Patient characteristics

Between November 25, 2020 and November 16, 2022, 17 patients were enrolled. Patient characteristics are shown in Table 3. The median age was 63 years old (range 47-76). Most participants were male (88.2%), Caucasian (88.2%), had stage IV disease (88.2%), and an Eastern Cooperative Oncology Group Performance Status (ECOG PS) of 1 (88.2%). Twelve patients had EAC (70.6%), 4 (23.5%) had GC, and 1 (5.9%) had GEJAC. The median number of prior lines of therapy was 2 (range: 1-4). (As noted in Table 3, the original version of the protocol that allowed patients with at least 1 prior line of therapy was amended to reflect a change in the expected objective response rate to single-agent irinotecan in the second-line setting).

**Table 3.** Patient characteristics.

**Number of patients, male and female**	Male, *n* = 15; female, *n* = 2
**Race**	White, *n* = 15; Native Hawaiian, *n* = 1; unknown, *n* = 1
**Ethnicity**	Hispanic or Latino, *n* = 3; not Hispanic or Latino, *n* = 14
**Age in years: Median (range)**	62 (47-76)
**Stage at study entry**	IV, *n* = 15; IIB, *n* = 1; missing, *n* = 1
**Number of prior systemic therapies: Median (range)**	2 (1*-4)
**Performance status: ECOG 0**	2
**Performance status: ECOG 1**	15
**Tumor type**	Esophageal, *n* = 12; gastric, *n* = 4; gastroesophageal junction, *n* = 1
**Differentiation**	Moderately differentiated, *n* = 2; moderately to poorly differentiated, *n* = 2; poorly differentiated, *n* = 7; undifferentiated, *n* = 1; other, *n* = 1; missing, *n* = 4

* After this trial was activated, a study^24^ was published that presented data demonstrating a lower response rate with single agent irinotecan in the third line setting than what was originally used to design this study. Therefore, the statistical analysis plan was amended to reflect the new evidence in the field. Subsequently, the new analysis plan also required a change in the eligibility to restrict enrollment to only those patients who have progressed on at least 2 lines of prior therapy. At the time the study protocol was amended, all patients enrolled except 1 had at least 2 prior lines of therapy.

## Primary assessment method

The primary endpoint was objective response, defined as a complete or partial response according to RECIST 1.1 response criteria (performed by either the treating investigator or independent review). Secondary endpoints include: disease control rate (DCR), time to progression (TTP), progression-free survival (PFS), and overall survival (OS) (Table 4). PFS is calculated as the time from the start of treatment to progression or death from any cause and OS is similarly defined as the point from treatment start date to death from any cause. DCR is calculated as the percentage of evaluable patients with best response as stable disease (SD), partial response (PR), or complete response.

**Table 4.** Primary assessment method.

**Title**	Objective response by RECIST 1.1 response criteria
**Number of patients enrolled**	17
**Numbers of patients evaluable for efficacy**	16
**Number of patients evaluated for safety**	17
**Evaluation method**	Complete or partial response by RECIST 1.1 response criteria
**Outcome notes**	See Table 1 for patient characteristics and Figures 1 and 2 for progression-free survival and overall survival curves, respectively

**Figure 1.**
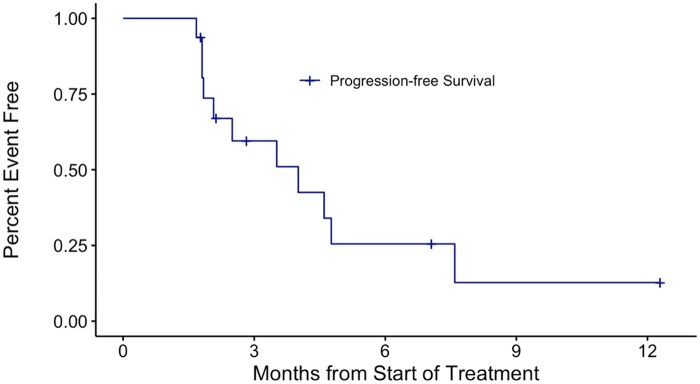
Progression-free survival (PFS) curve. The PFS curve represented in months from the start of treatment is shown. The median PFS was 4.01 months (95% CI: 2.07-not reached).

**Figure 2.**
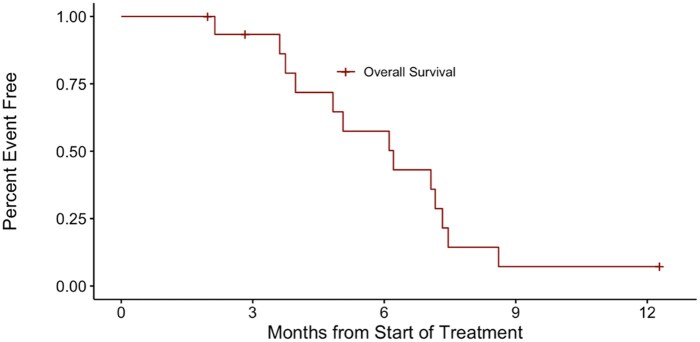
Overall survival (OS) curve. The overall survival curve is shown represented in months from the start of treatment. The median OS was 6.21 months (95% CI: 4.83-8.61).

All patients were off study treatment as of February 13, 2023 and off study as of October 11, 2023. One patient experienced clinical progression prior to the first response assessment and is not evaluable for response. No responses were observed in the 16 evaluable patients (ORR 0%; 95% CI 0%-19.4%). After the study had concluded enrollment and at the time the study database was reviewed for publication, we found a major deviation in the tumor response calculation. A patient with SD (−27% decrease) was incorrectly labeled as a PR, which was used in the Simon’s 2-stage design to continue to stage 2. This deviation was reported to CTEP and submitted to the NCI cIRB. Thus, the primary endpoint was not met. SD was observed in 9 of 16 patients, for a DCR of 56.2%. Median PFS was 4.01 months [95% CI: 2.07-not reached] (Figure 1). Median OS was 6.21 months [95% CI: 4.83-8.61] (Figure 2).

Prior response to platinum-based therapies was available for 14 patients. The mPFS for the sub−cohort analysis based on platinum sensitivity (sensitivity defined as PFS > 3 months) was 4.0 (95% CI: 1.7-4.8) months in the platinum-sensitive group (*n* = 10) and 1.9 (95% CI: 1.8-not reached) in the platinum-insensitive group (*n* = 4). The mOS for the platinum sensitive patients was 6.2 (95% CI: 3.7-7.2) months compared to 4.4 (95% CI: 2.1-NA) months in the platinum-insensitive group.

## Correlative studies

### Assessment of tumor DNA damage response

**Figure 3.**
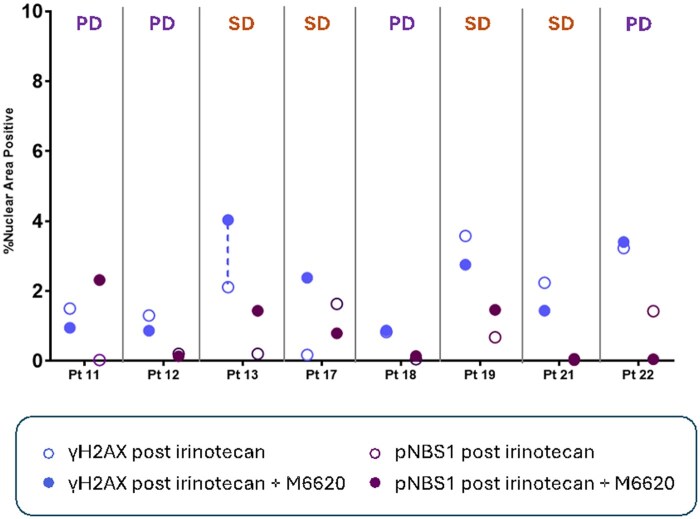
γH2AX and pNBS1 biomarker result summary. Modulation of DNA damage response markers post–irinotecan single agent and post–irinotecan and berzosertib combination treatment in patients with tumor analyzable at both time points. Percent NAP values for γH2AX (blue) and pNBS1 (pS343-NBS1, purple) are shown for tumor biopsies collected 21-27 hours post–irinotecan single agent treatment (open circle) and 21-27 hours post irinotecan and berzosertib combination treatment (closed circle). Patient responses to the treatment are shown above the graph. For patient 12 with stable disease (dotted line connecting data from 2 different time points), the signal for γH2AX biomarker was detected slightly above previously defined assay baseline of 4% nuclear area positive (NAP)^25^ in biopsies collected 21-27 hour post the end of irinotecan and berzosertib combination treatment.

**Figure 4.**
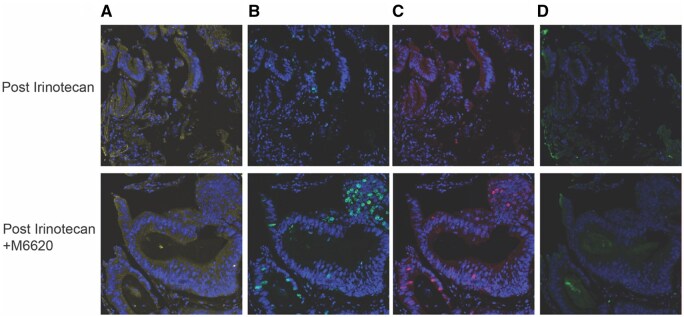
γH2AX, pNBS1, and pKAP1 immunofluorescence assays. Immunofluorescence assays for γH2AX (phosphorylated histone H2AX), pNBS1 (pS343-NBS1), and pKAP1 (pS824-KAP1) in paired tumor biopsies from patient 12 with stable disease. Biopsies were collected 21-27 hours post–irinotecan single agent treatment and 21-27 hours post–irinotecan and berzosertib (M6620) combination treatment. β-Catenin staining was used to demarcate tumor area for the analyses. Increases in γH2AX and pNBS1 levels were observed in the post combination treatment biopsy. (A) β-catenin, (B) γH2AX, (C) pNBS1, and (D) pKAP1.

The last 9 patients enrolled underwent pharmacodynamic biomarker biopsies at 21-27 hours post the end of irinotecan infusion and 21-27 hours post the end of irinotecan and berzosertib combination treatment to assess expression of DNA damage response biomarkers (γH2AX, pS343-NBS1, and pS824-KAP1) at both time points (Figures 3 and 4). We hypothesized that responding patients on study would have increased γH2AX post−berzosertib on cycle 2, day 2 since it accumulates at sites of DNA damage along with KAP1 p-Ser 824. Among 8 sets of evaluable biopsies, for 1patient with SD, the signal for γH2AX biomarker was detected slightly above previously defined assay baseline of 4% nuclear area positive (NAP)^25^ in biopsies collected 21-27 hour post the end of irinotecan and berzosertib combination treatment. This supports that combination treatment moderately increased the DNA damage response in this patient at this timepoint, although this may also reflect a false positive signal. According to a previous preclinical study,^26^ the peak of γH2AX biomarker response is evident at 4 hours post−topoisomerase I inhibitor treatment, after which the γH2AX response decreases. Therefore, the induction of γH2AX has passed the peak level in biopsies collected 21-27 hours post the end of irinotecan and berzosertib combination treatment. See Supplement 2 (see online supplementary material) for tissue collection, processing, and multiplex immunofluorescence assay methods.

## Adverse events

Treatment-related adverse events (TRAEs) observed in ≥10% patients are described in Table 5. The most common all-grade toxicities were gastrointestinal and hematologic. The most common ≥grade 3/4 TRAEs were neutropenia (4/17, 23.5% patients), anemia (3/17, 17.6%), febrile neutropenia (2/17, 11.8%), and diarrhea (2/17, 11.8%). There were no grade 5 AEs.

**Table 5.** Treatment-related adverse events in ≥ 10% patients.

Adverse event	Grade 1	Grade 2	Grade 3	Grade 4	Total
**Nausea**	5 (29.4%)	3 (17.6%)	1 (5.9%)	0 (0%)	9 (52.9%)
**Anemia**	0 (0%)	4 (23.5%)	3 (17.6%)	0 (0%)	7 (41.2%)
**Diarrhea**	4 (23.5%)	0 (0%)	3 (17.6%)	0 (0%)	7 (41.2%)
**Lymphocyte count decreased**	2 (11.8%)	2 (11.8%)	2 (11.8%)	1 (5.9%)	7 (41.2%)
**Fatigue**	4 (23.5%)	2 (11.8%)	0 (0%)	0 (0%)	6 (35.3%)
**White blood cell decreased**	3 (17.6%)	0 (0%)	1 (5.9%)	2 (11.8%)	6 (35.3%)
**Neutrophil count decreased**	2 (11.8%)	2 (11.8%)	1 (5.9%)	0 (0%)	5 (29.4%)
**Anorexia**	3 (17.6%)	0 (0%)	1 (5.9%)	0 (0%)	4 (23.5%)
**Vomiting**	2 (11.8%)	1 (5.9%)	1 (5.9%)	0 (0%)	4 (23.5%)
**Alopecia**	3 (17.6%)	0 (0%)	0 (0%)	0 (0%)	3 (17.6%)
**Dizziness**	3 (17.6%)	0 (0%)	0 (0%)	0 (0%)	3 (17.6%)
**Febrile neutropenia**	0 (0%)	0 (0%)	2 (11.8%)	1 (5.9%)	3 (17.6%)
**Hyponatremia**	2 (11.8%)	1 (5.9%)	0 (0%)	0 (0%)	3 (17.6%)
**Abdominal pain**	1 (5.9%)	1 (5.9%)	0 (0%)	0 (0%)	2 (11.8%)
**Alanine aminotransferase increased**	2 (11.8%)	0 (0%)	0 (0%)	0 (0%)	2 (11.8%)
**Alkaline phosphatase increased**	1 (5.9%)	1 (5.9%)	0 (0%)	0 (0%)	2 (11.8%)
**Aspartate aminotransferase increased**	2 (11.8%)	0 (0%)	0 (0%)	0 (0%)	2 (11.8%)
**Hypocalcemia**	1 (5.9%)	1 (5.9%)	0 (0%)	0 (0%)	2 (11.8%)
**Hypokalemia**	1 (5.9%)	1 (5.9%)	0 (0%)	0 (0%)	2 (11.8%)
**Sepsis**	0 (0%)	0 (0%)	2 (11.8%)	0 (0%)	2 (11.8%)

## Additional details of study design

### Study design

This study used a Simon’s 2-stage minimax design with a 1-sided type I error rate of 10% to evaluate the primary endpoint of ORR with a target ORR of 25%, relative to a historical control of patients treated with single agent irinotecan and estimated ORR of 5%. Evaluating 16 patients would provide at least 80% power to reject the null hypothesis that the ORR is ≤5% if the true ORR of the combination regimen is ≥ 25%. In the first stage of the trial, if 12 patients do not demonstrate a response, the trial will be stopped early for futility. The initial stage enrolled 6 safety lead-in patients and 6 additional patients, for a total of 12. If at least 1 patient experienced a response, then enrollment would continue to reach 16 efficacy-evaluable patients.

### Statistical analysis

Patient characteristics are reported descriptively using median and range for continuous variables and count with percentage for categorical variables. ORR, defined as the percentage of evaluable patients who experienced an objective response, was calculated along with 95% CIs. For both PFS and OS, medians and the corresponding 95% CI were calculated using the Kaplan−Meier Method. Frequencies and percentages of all TRAEs reported in ≥10% on the trial were summarized. All Grade 3+ adverse events were reported. No formal multiplicity adjustments were performed for secondary endpoints.

## Discussion

NCI 10211 was a phase II trial assessing the safety and efficacy of combination berzosertib and irinotecan in patients with *TP53*-mutated unresectable or metastatic GEC in the second- or later-line settings. Though there were no new safety signals, the study did not meet its primary endpoint as no responses were observed.

Patients with GEC have a poor prognosis, and *TP53* dysregulation leads to even shorter OS.^27^ Second- and third-line therapies in patients whose tumors do not have actionable mutations have historically demonstrated ORR between 20% and 30%^3^^,^^28–30^ and less than 5%,^31^ respectively. Later-line therapy to leverage DNA damage response mechanisms to augment the effect of chemotherapy in this cohort of patients remains challenging. The inclusion of 15 heavily pretreated patients (≥2 prior lines) likely contributed to the observed ORR falling below the pre−specified 25% threshold. Two patients were on study for 12.3 and 8.1 months, exceeding the typical PFS for patients receiving metastatic treatment in the first-line setting. These patients’ next-generation sequencing reports showed a tumor mutational burden of 7 mutations/megabase and a FANCI mutation, respectively, without other mutations in DNA-damage repair proteins. Additionally, the DCR of 56.2% is higher than typically seen in heavily pretreated patients where responses to irinotecan or docetaxel in later-line settings ranges from 38% to 53%.^32^ A study evaluating the efficacy of this combination using either DCR or PFS as a primary endpoint may provide further insight into the true efficacy in this molecularly selected patient population.

In addition to berzosertib, several ATR inhibitors have been studied in early phase trials. Ceralasertib, an oral ATR inhibitor, has been evaluated as monotherapy in a phase I study for advanced solid tumors (*n* = 66; upper gastrointestinal cancer, *n* = 7) and revealed a best overall response of SD in 52%, PR in 8%, and progressive disease (PD) in 41% in this heavily pretreated population (median 4 prior lines of therapy). Molecular data was available for 41 patients, 45% with a *TP53* alteration, and the authors found no correlation between *TP53* status and response or duration of response to ATR inhibition.^33^ Another potent oral ATR inhibitor, elimusertib, was similarly studied as monotherapy in patients with DNA damage repair deficiencies or ATM alterations (*n* = 143) refractory to standard therapies and found an ORR of 9% with DCR of 65% in tumors with ATM loss.^34^ Recently, data combining the novel, oral ATR inhibitor alnodersertib with irinotecan in patients with ATM-deficient solid tumors in the phase I/IIa STELLA trial (NCT04657068) revealed an ORR of 45% in patients with ATM-negative solid tumors.^35^ While awaiting longer-term data from this study, the early efficacy shows that ATR inhibitors may be more efficacious in patients with ATM loss or other DNA damage repair deficiencies.

Because ATR inhibitors have been shown to alter the tumor immune microenvironment by activating NK cells and increasing the number of tumor-infiltrating lymphocytes,^33^ a phase II trial was performed to study combination durvalumab (anti-PD-L1) and ceralasertib in patients with advanced GC in the second or later line (*n* = 31).^36^ Patients were not molecularly selected, and prior immunotherapy was allowed. In the 2 patients who had progressed on immunotherapy previously, 1 obtained a PR and 1, SD as best response. The ORR of the entire cohort was 22.6% with a DCR of 58.1% and mPFS of 3 months. In the subcohort of tumors with ATM loss and/or homologous repair deficiency, there was a significantly improved mPFS of 5.6 months compared to 1.7 months in the homologous repair proficient cohort (HR 0.13; *P* = 0.002).^36^

These data suggest that ATR inhibitors may be more efficacious when combined with immunotherapy or DNA-damaging chemotherapy agents in patients with ATM loss and/or DNA repair deficiencies, but less applicable in patients with *TP53* mutations. A *TP53* mutation is often inactivating^37^^,^^38^ and 1 *TP53* mutation may allow the cell’s other wild type p53 to increase in response to replicative stress. Several trials are ongoing to assess the efficacy of combination ATR and topoisomerase I inhibitors in advanced solid tumors with ATM loss (NCT06337630), combination ATR and PARP inhibition in solid tumors with DNA damage repair deficiency (NCT04972110), ATR and ATM inhibition in ATM loss of function (NCT05396833), and ATR inhibition with either PARP inhibitor or durvalumab (NCT03682289). These highly anticipated studies may change the treatment paradigm for DNA damage repair deficient tumors going forward. Though the combination of ATR inhibition with irinotecan was hypothesized to be synergistic in *TP53*-mutated GEC, this trial did not meet its primary endpoint and advances are needed to target *TP53* mutations going forward.

## Conclusion

Tumor mutation sequencing has led to the development and combination of novel therapies in patients with historically poor prognosis, including *TP53*-dysregulated disease. Though the combination of berzosertib and irinotecan was well tolerated, no responses were observed. Compared to historical controls in this setting, berzosertib and irinotecan demonstrated some activity and could be considered for further study in patients whose tumors harbor other DNA repair deficiencies. Continued innovative therapies are needed for patients with *TP53*-mutated, refractory disease to improve outcomes in this patient population.

## Supplementary Material

oyaf400_Supplementary_Data

## Data Availability

Aggregated data for this study will be published in Clinicaltrials.gov. Additional data will be shared on reasonable request to the corresponding author and approval by CTEP.
