# Supplementary material for: NCI 10211: a phase II, single-arm study of berzosertib in combination with irinotecan in patients with advanced TP53 mutant gastroesophageal cancer
Source: Oncologist. 2025 Dec 3;30(12):oyaf400. doi: 10.1093/oncolo/oyaf400 (PMC12718112; doi:10.1093/oncolo/oyaf400)
Supplement: oyaf400_Supplementary_Data [file oyaf400_supplementary_data.docx]

**Supplement 2: Correlative Studies Methods**

Human Tumor Biopsy Collection and Processing

Pairs of 18-gauge core-needle tumor biopsies were collected from patients enrolled in the study. Biopsies were obtained at 21-27 hours post the end of irinotecan infusion and 21-27 hours post the end of irinotecan and berzosertib combination treatment, then analyzed for levels of biomarkers γH2AX, pS343-NBS1, and pS824-KAP1 using the quantitative multiplex immunofluorescence assays validated for use on FFPE human tissue. All patients gave written informed consent for study participation. Study design and conduct complied with all applicable regulations, guidance, and local policies. Biopsies were placed in prechilled cryogenic vials, snap-frozen within 2 minutes of collection, and stored at ≤−80°C following DCTD standard operating procedures ([[SOP340507_RevI](https://dctd-cms.cancer.gov/drug-discovery-development/assays/validated-biomarker-assays/historical/sop340507-revi-ll-kfg.pdf))](https://dctd.cancer.gov/ResearchResources/biomarkers/docs/par/SOP340507_Biopsy_Frozen.pdf). Collected biopsies were fixed and paraffin blocked together with biomarker-positive control tissues for sectioning, following DCTD standard operating procedures ([[SOP340550: Tumor Frozen Needle Biopsy Preparation for Pharmacodynamic](https://dctd-cms.cancer.gov/drug-discovery-development/assays/validated-biomarker-assays/ncln/sop340550-biopsy-section-testis-jejunum-controls.pdf))](https://dctd.cancer.gov/ResearchResources/biomarkers/DDR3/SOP340550_Biopsy_Section_Testis_Jejunum_Controls.pdf).

Multiplex Immunofluorescence Assays

The staining of FFPE tissue sections using the BOND RX autostainer was performed following DCTD standard operating procedures ([[LHTP003.0723_Biopsy_BondRX_gH2AX_pNBS1_pKAP1_BCat](https://dctd-cms.cancer.gov/drug-discovery-development/assays/validated-biomarker-assays/ncln/lhtp003.07.23-biopsy-bondrx-gh2ax-pnbs1-pkap1-bcat.pdf))](https://dctd.cancer.gov/ResearchResources/biomarkers/DDR3/SOP340543_Biopsy_BondRX_gH2AX_pNBS1_BCat.pdf); detailed BOND RX System (Leica Biosystems) methods can be obtained from the manufacturer. For γH2AX and pS343-NBS1 analysis, biotin-conjugated γH2AX (clone JBW301; EMD Millipore) and a custom-conjugated pS343-NBS1-DIG (clone EP178; Abcam) antibodies were detected with the use of Streptavidin labeled with Alexa Fluor 488 and Alexa Fluor 647 custom-conjugated IgG Fraction Monoclonal Mouse Anti-Digoxin antibody, respectively. For pS824-KAP1 analysis, anti- pS824-KAP1 antibody (clone EPR5248; Abcam) custom conjugated to Dinitrophenol (DNP; Molecular Probes, Inc.) was detected using Alexa Fluor 488 conjugated anti-Dinitrophenyl KLH Rabbit Polyclonal antibody. In addition, β-Catenin antibody custom conjugated to Alexa Fluor 546 was used to define tumor areas in all biomarker analyses. After staining with a cocktail of the primary antibodies, followed by washing and staining with the secondary reporters, the slides were rinsed in BOND Wash Solution and blotted to remove excess liquid. Slides were cured overnight protected from light with Prolong Gold Antifade Reagent (Invitrogen) and imaged the following day. For γH2AX and pS343-NBS1 analysis, fluorescent whole slide images were acquired at 20X using Leica’s Aperio FL scanner. Images were then analyzed using a custom algorithm build in Definiens Tissue Studio Analysis software (formerly Definiens AG) following published DCTD standard operating procedures [SOP340545: Image Extraction and Analysis of Tumor Biopsy Slides from gH2AX, pNBS1 IFA with b-Catenin Segmentation](https://dctd-cms.cancer.gov/drug-discovery-development/assays/validated-biomarker-assays/ncln/sop340545-image-analyze-gh2ax-pnbs1-bcat.pdf)). For pS824-KAP1 analysis, whole slide fluorescent images were acquired at 20X using ZEISS Axioscan 7 scanner. pKAP1 biomarker signals were qualitatively evaluated.
